# Intraoperative functional zero-position is associated with superior outcomes after reverse total shoulder arthroplasty for cuff tear arthropathy: a retrospective comparative study

**DOI:** 10.1016/j.jsea.2026.100033

**Published:** 2026-04-29

**Authors:** Ryo Tazawa, Tomonori Kenmoku, Mitsufumi Nakawaki, Daisuke Ishii, Mitsuyoshi Matsumoto, Kosuke Inoue, Tai Yoshizawa, Yuta Konno, Gen Inoue, Masashi Takaso

**Affiliations:** Department of Orthopaedic Surgery, Kitasato University School of Medicine, Sagamihara, Kanagawa, Japan

**Keywords:** Reverse total shoulder arthroplasty, Cuff tear arthropathy, Functional zero-position, Soft-tissue balance, Fluoroscopy, Range of motion, Clinical outcomes

## Abstract

**Background:**

Optimal implant positioning in reverse total shoulder arthroplasty (rTSA) remains challenging because of the lack of standardized intraoperative criteria. The “zero-position,” defined as alignment of the humeral axis with the scapular spine, represents a balanced state in native shoulders. In rTSA, a similar alignment, termed the functional zero-position, may reflect balanced glenohumeral motion. This study evaluated its association with post-operative outcomes in patients with cuff tear arthropathy (CTA).

**Methods:**

A retrospective review of 45 shoulders (43 patients) with CTA and ≥2-year follow-up was performed. Patients were grouped based on intraoperative achievement of the functional zero-position under fluoroscopic guidance during passive scaption. Range of motion and Constant Murley scores were compared. Fisher exact test, Mann–Whitney *U* test, Friedman test, and two-way repeated-measures analysis of variance were used (*P*< .05).

**Results:**

The functional zero-position was achieved in 35 shoulders. Both groups showed significant improvement in flexion and abduction from 6 months onward. A significant group-by-time interaction was observed for elevation (flexion, *P*= .0024; abduction, *P*= .0005). Flexion was significantly greater in the functional zero-position group from 6 months, and abduction after 1 year. External rotation improved only in the functional zero-position group, with significant between-group differences beginning at 6 months. At final follow-up, elevation, external rotation, and Constant Murley scores were higher in the functional zero-position group.

**Conclusion:**

In CTA, intraoperative achievement of the functional zero-position was associated with superior range of motion, favorable recovery, and improved function, suggesting that it serves as a practical intraoperative reference for optimizing soft-tissue balance in rTSA.

Reverse total shoulder arthroplasty (rTSA) is a well-established treatment for cuff tear arthropathy (CTA) and rotator cuff-deficient shoulders, providing effective pain relief and functional restoration. The altered biomechanics following rTSA, including medialization and a downward shift of the center of rotation, enhance deltoid tension, creating a more stable fulcrum.^1^^,^^4^^,^^8^ However, these biomechanical changes require careful attention to soft-tissue balance, which is critical for post-operative stability and range of motion (ROM). Despite its importance, optimal soft-tissue balance remains undefined because it depends on complex anatomical, biomechanical, and surgical factors.^8^^,^^22^ In recent years, surgical assistance technologies such as navigation and robot-assisted systems have improved the accuracy and reproducibility of implant positioning in shoulder arthroplasty.^2^^,^^25^^,^^30^ Moreover, changes in scapulohumeral rhythm after rTSA have recently attracted attention because they may provide further insights into post-operative shoulder function.^11^^,^^15^ However, despite these technological and biomechanical developments, no universally accepted intraoperative criterion exists for achieving optimal soft-tissue balance in rTSA.

The “zero-position,” defined as alignment of the humeral axis with the scapular spine, represents a biomechanically balanced state that minimizes muscular forces during shoulder elevation.^5^^,^^23^^,^^24^ In native shoulders, this position is associated with enhanced joint stability and function. Codman^5^ described it as a pivotal point at which the deltoid and rotator cuff muscles are relaxed, promoting favorable tissue repair conditions. Saha^24^ further highlighted its stability, noting the minimal muscular rotatory forces required to maintain this position. Although rTSA substantially alters shoulder biomechanics, a similar alignment observed during passive elevation may still represent a balanced state of glenohumeral motion. In the present study, we define this intraoperative dynamic alignment in rTSA as the functional zero-position. This allows us to distinguish it from Saha's original concept and to emphasize its role as a functional intraoperative indicator rather than a static anatomical position. However, the clinical relevance of this functional zero-position in rTSA remains unclear.

This retrospective study aimed to evaluate the clinical utility of the functional zero-position as an intraoperative indicator for implant positioning and soft-tissue balance in rTSA. We hypothesized that intraoperative achievement of the functional zero-position would be associated with superior post-operative ROM and functional outcomes in patients with CTA.

## Materials and methods

This study was designed as a Level III retrospective cohort study and was conducted following approval from the institutional review board (KMEO B19-062). All patients provided informed consent for both the surgical procedure and the use of their clinical data for research purposes.

### Patients

Between October 2014 and December 2023, consecutive rTSA cases were screened. Inclusion criteria were: (1) diagnosis of CTA, (2) primary rTSA, and (3) ≥2 years of follow-up. To minimize confounding from diagnostic heterogeneity, only patients with CTA were included. The pre-operative status of the rotator cuff, including the subscapularis and teres minor tendons, was assessed using pre-operative magnetic resonance imaging. Exclusion criteria included neurological paralysis. A total of 45 shoulders (43 patients) met criteria. The mean age was 78.2 (range, 67-91) years, with 13 men and 32 women. Thirty-one shoulders were on the right side and 14 on the left side.

**Figure 1:**
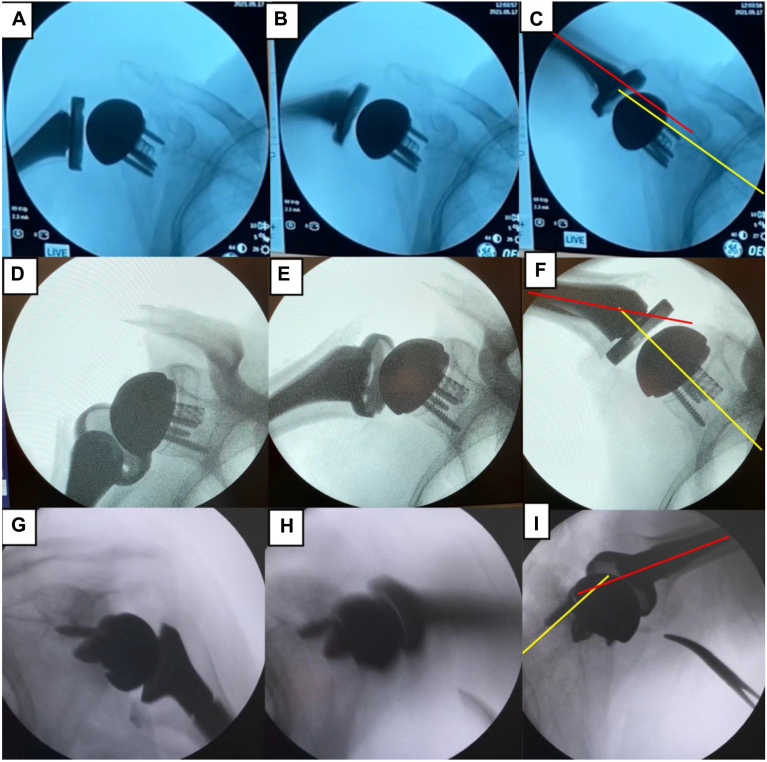
Intraoperative fluoroscopic images comparing passive scaption motion between the functional zero-position (**A-C**) and the nonfunctional zero-position (**D-I**) groups (**A**, **D**, and **G**): early phase; (**B**, **E**, and **H**): mid-range phase; (**C**, **F**, and **I**): maximal scaption. (**C**) demonstrates the achievement of the functional zero-position. (**F**) illustrates subacromial bony impingement, whereas (**G**) shows scapula-dominant scaption. The *red solid line* represents the humeral axis, and the *yellow dashed line* indicates scapular spine alignment.

Patients were divided into 2 groups based on whether the functional zero-position was achieved intraoperatively during passive scaption under fluoroscopy. The patient's arm was elevated in the scapular plane by grasping the wrist. Shoulders achieving the functional zero-position intraoperatively (Fig. 1, A-C, Video 1) were classified as the functional zero-position group, whereas those failing to do so (Fig. 1, D-I, Video 1) were classified as the nonfunctional zero-position group.

### Implant details

Implant variables, including manufacturer, stem retroversion angle, and glenosphere size, were analyzed to assess their impact on post-operative outcomes across multiple implant systems.

### Clinical assessments

ROM assessments (active flexion, abduction, external rotation, and internal rotation) were conducted pre-operatively and at 3 months, 6 months, 1 year, 2 years, and at final follow-up. Internal rotation was measured as the highest spinal level reached with the thumb. The Constant Murley score was recorded pre-operatively, at 2 years post-operatively, and at the final follow-up.^6^

### Surgical procedure

All surgeries were performed under general anesthesia in the beach-chair position. Interscalene blocks were administered when feasible. A deltopectoral approach was consistently used, with subscapularis peeling and reattachment performed when intact.

Standard surgical techniques and instrumentation were followed according to the implant manufacturer's guidelines. Humeral retroversion was determined intraoperatively by flexing the elbow to 90° using a retroversion guide attached to the humeral stem holder and aligning it with the forearm axis to guide humeral stem insertion. All components were implanted without cement. Post-operatively, patients were placed in a sling for 4 weeks. Passive ROM exercises began on post-operative day 1, and active ROM was initiated at 4 weeks.

### Functional zero-position assessment

After rTSA placement, the patient's arm was passively elevated in the scapular plane through full available scaption under general anesthesia by grasping the wrist. Fluoroscopic evaluation was performed using anteroposterior images. The C-arm was rotated until the glenoid articular surface was in profile and the glenohumeral joint centered, and this alignment was maintained after rTSA placement. Under these conditions, functional zero-position was defined as alignment of the humeral axis with the scapular spine during passive scaption in the scapular plane (Fig. 1, Video 1). If functional zero-position could not be achieved, the surgeon evaluated possible causes such as excessive deltoid tension or subacromial bony impingement. When indicated, intraoperative adjustments included modifying humeral stem height, altering the humeral osteotomy level, or changing the humeral stem retroversion angle to restore glenohumeral motion and facilitate functional zero-position achievement. If soft-tissue contracture was identified, soft-tissue release was performed to the extent possible. In these cases, stability after rTSA placement was prioritized over achieving functional zero-position.

### Intraoperative motion assessment

Intraoperative scaption patterns were qualitatively assessed by fluoroscopic observation.

“Scapula-dominant scaption” was defined as marked scapular motion beginning in the early phase of passive scaption. In contrast, in the functional zero-position group, humeral elevation was initiated first, followed by scapular motion.

“Subacromial bony impingement” was defined as mechanical obstruction during elevation caused by contact between the greater tuberosity and the acromion, resulting in restricted smooth motion.

### Statistical analysis

Statistical analyses were conducted using SPSS version 27 (IBM Corp., Armonk, NY, USA). Fisher exact test was used for categorical variables and the Mann–Whitney *U* test for between-group comparisons of continuous variables. Within-group changes in ROM and Constant Murley scores over time were analyzed using the Friedman test, followed by post hoc Wilcoxon signed-rank tests when appropriate. Furthermore, a two-way repeated-measures analysis of variance was performed to evaluate group-by-time interactions for post-operative ROM. Statistical significance was set at *P*< .05.

## Results

Thirty-five shoulders were classified as the functional zero-position group and 10 as the nonfunctional zero-position group. In the nonfunctional zero-position group, 2 distinct movement patterns were identified on intraoperative fluoroscopy, as defined in the Methods: scapula-dominant scaption and subacromial bony impingement (Fig. 1, D-I, Video 1).

### Characteristics and surgical details

The demographic characteristics and surgical details are summarized in Table I. Although participants in the functional zero-position group tended to be younger than those in the nonfunctional zero-position group, this difference did not reach statistical significance (*P*= .069). There were no significant differences in sex distribution or operated side between the groups. There were also no significant differences in the pre-operative status of the subscapularis and teres minor tendons between the groups.

**Table I:** Clinical characteristics of patients.

Characteristic	Functional zero-position (n = 35)	Nonfunctional zero-position (n = 10)	*P* value
Mean age (yr)	77.3 ± 4.7	81.1 ± 5.6	.069
Sex (male, female)	11, 24	2, 8	.482
Height (cm)	152.9 ± 8.7	148.5 ± 5.7	.131
Weight (kg)	57.6 ± 12.2	50.9 ± 8.2	.162
Operated side, n (right, left)	24, 11	7, 3	.931
Follow-up duration (mo)	38.5 ± 18.8	39.1 ± 16.1	.619
Subscapularis tear, n (%)	17 (48.5)	4 (40.0)	.729
Teres minor tear, n (%)	4 (11.4)	1 (10.0)	1.000

### Implant details

Implant-related details, including implant design, stem retroversion angle, and glenosphere size, are presented in Table II. No significant differences were observed between the 2 groups for these parameters.

**Table II:** Implant details.

Characteristic	Functional zero-position (n = 35)	Nonfunctional zero-position (n = 10)	*P* value
Implant manufacturer	Ex, 25; En, 3; S, 1; Z, 6	Ex, 6; En, 2; S, 1; Z, 1	.530
Implant design (inlay, onlay)	4, 31	4, 6	.059
Stem retroversion (°)	13.6 ± 8.6	17.5 ± 5.7	.111
Glenosphere (mm)	36.7 ± 1.4	36.7 ± 1.1	.443

*Ex*, Exactech; *En*, Enovis; *S*, Stryker; *Z*, Zimmer Biomet.

### Post-operative outcomes

**Figure 2:**
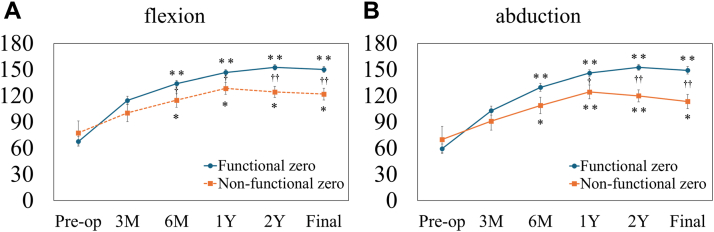
Pre-operative and post-operative elevation angles in flexion (**A**) and abduction (**B**). Recovery trajectories of flexion and abduction differ significantly between groups. ∗*P*< .05, ∗∗*P*< .001 vs. pre-operative; †*P*< .05, ††*P*< .001 between groups in (**A**). ∗*P*< .05, ∗∗*P*< .001 vs. pre-operative; †*P*= .012, ††*P*< .001 between groups in (**B**).

**Figure 3:**
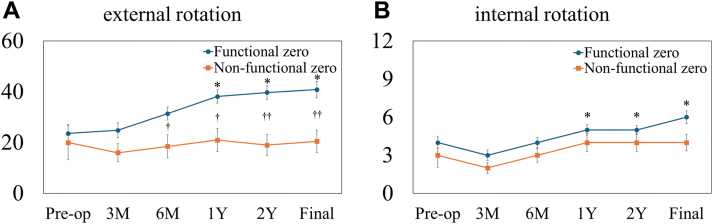
Pre-operative and post-operative rotation angles in external rotation (**A**) and internal rotation (**B**). ∗*P*< .001 vs. pre-operative; †*P*< .05, ††*P*< .01 between groups in (**A**). ∗*P*< .001 vs. pre-operative in (**B**).

**Figure 4:**
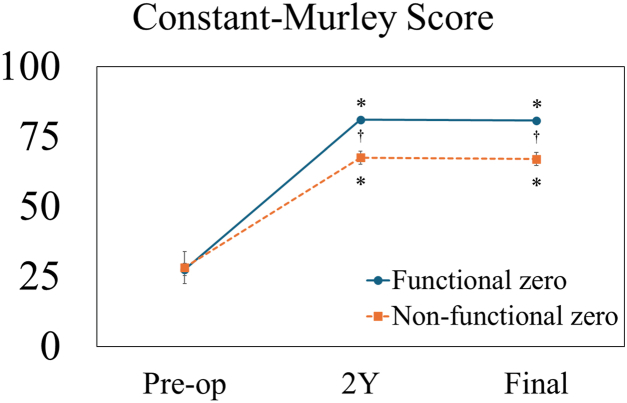
Pre-operative and post-operative Constant Murley scores. ∗*P*< .001 vs. pre-operative, †*P*< .001 between groups.

Post-operative outcomes, including ROM and Constant Murley scores, are shown in Figure 2, Figure 3, Figure 4.

Both groups demonstrated significant improvements in elevation (flexion and abduction) from 6 months post-operatively onward compared with pre-operative values (*P*< .001 for all). A significant group-by-time interaction was observed for elevation (*P*= .0024 for flexion; *P*= .0005 for abduction), indicating different recovery trajectories between groups. Accordingly, the functional zero-position group showed significantly greater flexion from 6 months post-operatively onward and greater abduction from 1 year onward compared with the nonfunctional zero-position group (*P*< .05 for all).

External rotation showed significant improvement in the functional zero-position group from 1 year post-operatively onward compared with pre-operative values (*P*< .001 for all). No significant improvement was observed in the nonfunctional zero-position group. External rotation was significantly greater in the functional zero-position group than in the nonfunctional zero-position group from 6 months post-operatively onward (*P*< .05 at 6 months and 1 year, *P*< .01 at 2 years, and final follow-up). Internal rotation demonstrated significant improvement in the functional zero-position group, with earlier improvements observed from 1 year post-operatively and sustained across all subsequent follow-up time points compared with pre-operative values (*P*< .001 at 1 year, 2 years, and at final follow-up). In the nonfunctional zero-position group, internal rotation did not show a significant improvement compared with pre-operative values. No significant difference in internal rotation was observed between the 2 groups at any post-operative time point. No significant group-by-time interaction was observed for rotation (*P*= .051 for external rotation; *P*= .73 for internal rotation).

The Constant Murley scores showed significant improvement in both groups at 2 years post-operatively and at the final follow-up compared with pre-operative values (*P*< .001 for all). Between-group comparisons showed that the functional zero-position group demonstrated significantly higher Constant Murley scores than the nonfunctional zero-position group at 2 years post-operatively and the final follow-up (*P*< .001 for both).

## Discussion

Our study established that intraoperative attainment of the functional zero-position was significantly associated with enhanced post-operative ROM, particularly in elevation and external rotation, as well as higher Constant Murley scores. In addition to superior final post-operative ROM, the present study demonstrated that the time course of recovery differed significantly between groups. The functional zero-position group showed significantly greater flexion from 6 months post-operatively and greater abduction from 1 year onward compared with the nonfunctional zero-position group. In contrast, the nonfunctional zero-position group exhibited a more limited recovery trajectory despite overall post-operative improvement. These findings suggest that intraoperative achievement of the functional zero-position is associated not only with a greater final magnitude of recovery but also with a more favorable pattern and quality of post-operative glenohumeral motion restoration. Collectively, these results highlight the importance of intraoperative soft-tissue balance and support the functional zero-position as a practical intraoperative reference for optimizing functional outcomes after rTSA.

The functional success of rTSA is heavily dependent on the performance of the deltoid muscles, which is essential for maintaining shoulder stability and generating force.^4^ However, the biomechanics of the rTSA shoulder differ markedly from a normal shoulder, exhibiting a greater scapulohumeral rhythm and augmented upper trapezius activity in addition to deltoid engagement.^28^^,^^29^ Studies have shown that patients with slouched postures exhibit a reduced ROM in the rTSA compared with those with a more neutral posture.^21^ Furthermore, shoulders exhibiting poor elevation post-operatively demonstrated greater glenosphere upward rotation than those achieving optimal elevation after rTSA.^16^ These reports reinforce that glenohumeral joint mobility is crucial for optimal elevation after rTSA, although scapular motion is often greater than that in the normal shoulder. Achieving the functional zero-position intraoperatively ensures the preservation of glenohumeral motion following rTSA. Consequently, improvements in ROM are largely contingent on the functionality of the deltoid muscles. In our study, the clinical improvements in ROM were sustained throughout the follow-up period in the functional zero-position group. This suggests that intraoperative confirmation of the functional zero-position could be a practical and effective criterion for optimizing implant positioning, ensuring favorable post-operative outcomes in rTSA. Securing smooth glenohumeral motion and avoiding subacromial bony impingement during surgery are critical for achieving the best results.

Conversely, patients who failed to achieve the functional zero-position intraoperatively exhibited poorer post-operative outcomes. In the nonfunctional zero-position group, scapula-dominant scaption was frequently observed, defined intraoperatively as excessive scapular motion relative to the humerus during elevation, disrupting normal glenohumeral motion. Previous studies have highlighted the importance of preserving glenohumeral motion; one identified intraoperative forward flexion as the strongest predictor of post-operative ROM, indicating that greater forward flexion reflects less soft-tissue tightness and more favorable glenohumeral mobility.^26^ This highlights the value of intraoperative motion assessments in evaluating soft-tissue balance. Achieving good intraoperative motion is therefore critical for favorable post-operative outcomes.

Post-operatively, the period from 6 months onward appears to be critical for functional recovery of glenohumeral motion.^17^^,^^27^ Consistent with these observations, in our study, both groups showed significant improvements in elevation from 6 months post-operatively; however, the functional zero-position group consistently achieved superior elevation outcomes thereafter. In contrast, recovery in the nonfunctional zero-position group was characterized by persistently inferior post-operative motion despite similar timing of improvement. These findings suggest that intraoperative achievement of the functional zero-position is associated with a more favorable quality of post-operative glenohumeral motion recovery.

Recent reports have similarly shown that poor early clinical outcomes, particularly low American Shoulder and Elbow Surgeons scores within the first 3-6 months after rTSA, are strongly associated with persistent functional limitations at later follow-up, highlighting the prognostic importance of the early post-operative phase.^9^ This highlights the impact of intraoperative positioning on glenohumeral motion recovery, which is essential for optimizing post-operative outcomes. Consistent with these observations, the present findings suggest that intraoperative achievement of the functional zero-position is associated with a more favorable recovery pattern of glenohumeral motion, as reflected by superior elevation and external rotation from 6 months post-operatively onward. This indicates that intraoperative positioning may influence the quality of post-operative recovery during this critical period.

Furthermore, kinematic analyses have shown that favorable elevation outcomes are associated with increased scapulohumeral rhythm and sufficient glenohumeral motion, whereas poor outcomes are often associated with compensatory scapular movement.^16^ One significant factor that impedes smooth glenohumeral motion is the risk of increased deltoid tension resulting from arm lengthening, which has been shown to increase joint load, impair muscle efficiency, and negatively impact post-operative function.^7^ Specifically, arm lengthening exceeding 2.5 cm relative to the contralateral side is particularly concerning^13^ because it can lead to complications such as neurologic damage or acromial fracture.^12^^,^^14^ To mitigate these risks, we have adopted a technique involving sufficient humeral osteotomy, performed distally just above the remaining external rotator cuff, thereby preventing over-tightening of the deltoid. This approach ensures smooth glenohumeral motion and reduces complications related to overtightened soft tissues.

Another characteristic finding in the nonfunctional zero-position group was subacromial bony impingement, as defined in the Methods, representing a mechanical obstruction caused by contact between the greater tuberosity and the acromion during elevation. A previous study reported that subacromial notching occurred in 12.8% of rTSA cases and was associated with poorer post-operative outcomes.^10^ Dynamic analysis has shown that external rotation of 5-10° during elevation is essential for smooth shoulder motion.^18^ These findings highlight the critical importance of achieving adequate external rotation to minimize the risk of subacromial impingement and its complications. In contrast, pre-operative ROM simulations are typically based on motion along a single axis and do not fully capture the coupled rotational movements of the shoulder, such as external rotation during elevation. Therefore, intraoperative dynamic assessment may provide a more physiologically relevant evaluation of functional motion, supporting the clinical relevance of intraoperative assessment in the present study. In cases of severe atrophy of the infraspinatus and teres minor, we opted to decrease the retroversion of the humeral stem insertion, allowing external rotation early in the ROM and reducing the risk of subacromial bony impingement while supporting improved shoulder function.^20^ Although not statistically significant, the functional zero-position group tended to show smaller retroversion angles, which may reflect this intraoperative strategy and could have facilitated achievement of the functional zero-position. Further studies are needed to validate this potential association.

Previous studies have reported that older age and shorter stature are associated with inferior outcomes after rTSA,^3^^,^^17^^,^^19^ potentially due to increased deltoid tension, arm lengthening, and a reduced subacromial space, particularly when humeral osteotomy is performed at the anatomical neck level. These factors may disrupt smooth glenohumeral motion and increase the risk of subacromial impingement. In the present CTA-only cohort, however, no significant differences in age or height were observed between the functional zero-position and nonfunctional zero-position groups, although the nonfunctional zero-position group tended to be older and shorter. Given the limited sample size, the influence of age and stature on functional zero-position achievement could not be definitively determined. Nevertheless, these patient-related factors remain clinically relevant considerations, and particular attention to the humeral osteotomy level, deltoid tension, and maintenance of sufficient external rotation may be especially important in older or small-stature patients to facilitate smooth glenohumeral motion and avoid subacromial impingement during rTSA. Considering this interindividual variability, it may be difficult to define uniform surgical parameters applicable to all patients. In this context, the intraoperative achievement of the functional zero-position may serve as an integrated indicator reflecting the overall balance between implant configuration and soft-tissue tension.

### Strengths and limitations

This study has several strengths. First, it proposes the functional zero-position as a practical and straightforward intraoperative criterion for assessing implant positioning and soft-tissue balance in rTSA. Assessment of the functional zero-position can be performed intraoperatively using standard fluoroscopy, making it readily applicable in routine clinical practice. The present findings demonstrate a clear association between intraoperative achievement of the functional zero-position and improved post-operative ROM and functional outcomes in patients with CTA, supporting its clinical relevance as a simple and reproducible intraoperative reference. This approach provides a more objective and standardized method of intraoperative assessment, thereby reducing surgeon-dependent variability.

Second, post-operative outcomes were comprehensively evaluated using both ROM and Constant Murley scores at multiple time points. This longitudinal assessment allowed detailed characterization of not only final outcomes but also temporal recovery patterns, including early, midterm, and final post-operative phases. Such time-course analysis strengthens the interpretation of the relationship between intraoperative findings and post-operative functional recovery.

However, this study also has several limitations. Owing to its retrospective design, a causal relationship between intraoperative achievement of the evaluated functional zero-position and post-operative outcomes cannot be definitively established. Future studies should therefore adopt a prospective design in which intraoperative assessment is predefined and standardized. Although the present study focused exclusively on patients with CTA to minimize diagnostic heterogeneity, the specific mechanisms underlying failure to achieve this intraoperative position were not quantitatively assessed. Intraoperatively, failure to achieve this position was consistently associated with impaired glenohumeral motion and mechanical constraints, particularly subacromial bony impingement; however, these findings were based on qualitative assessment without objective measurements. Factors such as deltoid tension, soft-tissue contracture, and implant-related parameters, including humeral osteotomy level, stem height, and humeral stem retroversion, were evaluated clinically during surgery but were not measured using objective or quantitative indices. These observations suggest that reduced glenohumeral motion combined with increased mechanical resistance may play a central role in limiting functional elevation. This represents an important limitation of the present study. Future investigations incorporating direct intraoperative measurements of soft-tissue tension and quantitative evaluation of glenohumeral and scapulothoracic motion may help to better elucidate the mechanisms underlying this intraoperative finding and further develop this concept. Furthermore, the relatively small sample size may limit the statistical power of the analysis and the generalizability of the findings, particularly in the context of variability in implant design. Finally, although the mean follow-up duration was sufficient to evaluate short- to midterm clinical outcomes, it did not provide insights into long-term results. Further studies with longer follow-up periods are required to determine whether the benefits associated with intraoperative functional zero-position achievement are sustained over time.

In the absence of universally accepted intraoperative criteria for optimizing implant positioning and soft-tissue balance in rTSA, confirmation of the functional zero-position under fluoroscopy may provide a simple and practical intraoperative reference for achieving appropriate deltoid tension and smooth glenohumeral motion, ultimately contributing to improved surgical outcomes in CTA.

## Conclusion

In patients with CTA, intraoperative achievement of the functional zero-position during rTSA was associated with superior post-operative ROM and improved functional outcomes, as well as more favorable recovery trajectories of shoulder elevation over time. Assessment of the functional zero-position provides a simple intraoperative indicator of adequate soft-tissue balance and smooth glenohumeral motion without subacromial bony impingement. Incorporating confirmation of the functional zero-position into intraoperative decision-making may facilitate favorable recovery of shoulder motion and contribute to optimized implant placement and improved clinical outcomes in rTSA.

## Disclaimers

Funding: No funding was disclosed by the authors.

Conflicts of interest: The author, their immediate family, and any research foundation with which they are affiliated have not received any financial payments or other benefits from any commercial entity related to the subject of this article.

## Declaration of generative AI and AI-assisted technologies in the writing process

AI-assisted tools (ChatGPT, OpenAI, GPT-5.3) were used solely to improve the clarity of English expression and to assist with formatting of the abstract and response to reviewers.

## Supplementary data

Video 1 Intraoperative fluoroscopic assessment of the functional zero-position during reverse shoulder arthroplasty. This video first demonstrates the intraoperative method for evaluating functional zero-position achievement. The patient's arm is passively elevated in the scapular plane through full available scaption by grasping the wrist. Fluoroscopic anteroposterior imaging is then used to determine functional zero-position achievement, defined as alignment of the humeral axis with the scapular spine. The video subsequently illustrates 3 distinct patterns of passive scaption observed intraoperatively: (1) functional zero-position achievement characterized by smooth glenohumeral motion without excessive scapular movement or impingement; (2) scapula-dominant scaption marked by excessive scapular motion and disrupted glenohumeral kinematics; and (3) subacromial bony impingement caused by mechanical contact between the greater tuberosity and the acromion.
